# Items

**Published:** 1900-08

**Authors:** 


### ITEMS.

THE *Medical Mirror*, of St. Louis, offers $1,000 in prizes for essays on tuberculosis, to be distributed as follows: $500, $200, $100, and four prizes of $50 each. The following prominent gentlemen have accepted appointment on the committee on awards: Dr. Wm. Osler, Baltimore, Md.; Dr. George F. Butler, Chicago, Ill.; Dr. A. R. Kiefer, St. Louis, Mo.; Dr. C. Lester Hall, Kansas City, Mo.; Dr. H. R. Hall, St. Louis, Mo.; Dr. Lewis E. Lemen, Denver, Col.; Dr. Jos. M. Mathews, Louisville, Ky.; Dr. W. W. Grant, Denver, Col.; Dr. Thos. Hunt Stucky, Louisville, Ky.; Dr. Hugo Summa, St. Louis, Mo.; Dr. Walter Wyman, Washington, D.C. Entries close October 1, 1900, and the award is to be made January 1, 1901, giving the contestants three months in which to prepare their papers and to include clinical reports. The points for consideration in each paper with a percentage attached will be as follows: General considerations of the subject, 10; pathology, bacteriology and diagnosis, 20; clinical reports, 20; prognosis and treatment, 35; conclusions with résumé, 15. The *Mirror* hopes to develop from this discussion the fact that much more can be done for tubercular patients than the average practitioner believes, and is aiming to elicit all facts that may be of value in the treatment of this disease.

---

Messrs. Battle & Co., of St. Louis, are sending out a picture illustrating the tortures of the gout. It is a copy of a drawing published May 14, 1799, by H. Humphrey, 27 St. James Street, London. To be appreciated it must be seen. The publishers will gladly send the engraving to any physician who may request the same by addressing a line to Messrs. Battle & Co., Chemist Corporation, 2001 Locust Street, St. Louis.

---

Messrs. Reed & Carnrick have recently added complete physiological, chemical, pathological and bacteriological laboratories to their plant at 42 to 46 Germania Avenue, Jersey City, N.J. The members of the American Medical Association on their way home from the Atlantic City meeting were invited to visit these laboratories. Those who accepted were received by Dr. Warner, who with his assistants showed the visitors through the several rooms and explained the work that was being done in them. The apartments are tiled in white and are supplied with all apparatus and instruments necessary to do accurate work in physiology, pathology, chemistry and bacteriology. The complete accessories, microscopic instruments, incubators, etc., are of the best make, and were furnished by the reliable and old-established firm of Queen & Co., of Philadelphia, Pa. The profession is invited to submit specimens and to investigate the laboratories. Fees for examination are very moderate; they may be had on application. Physicians passing through Jersey City and having the spare time to take a few minutes' ride on the Turnpike trolley may spend a delightful hour at the laboratories.

---

Attention is invited to advertising p. xxx., on which will be found two "For Sale" notices that will interest physicians either in search of a location or in want of an outfit, such as books, instruments and office furniture.

---

Notice.—All Surgeons, Assistant Surgeons, Acting Assistant Surgeons or Contract Surgeons, and Hospital Stewards, who served in the Army or Navy of the late Confederate States, will please send their post-office address to Deering J. Roberts, M.D., Secretary Surgeons' Association, C.S.A., Nashville, Tenn.

